# Pharmacological strategies used to manage symptoms of patients dying of COVID-19: A rapid systematic review

**DOI:** 10.1177/02692163211013255

**Published:** 2021-05-13

**Authors:** Laura Heath, Matthew Carey, Aoife C Lowney, Eli Harriss, Mary Miller

**Affiliations:** 1 Nuffield Department of Primary Care Health Sciences, University of Oxford, Oxford, UK; 2 Oxford University Hospitals NHS Foundation Trust, Sir Michael Sobell House Hospice, Oxford, UK; 3 Bodleian Health Care Libraries, University of Oxford, Oxford, UK; 4 Nuffield Department of Medicine, University of Oxford, Oxford, UK

**Keywords:** COVID-19, coronavirus, SARS-CoV-2, systematic review, palliative care, palliative medicine, symptom management, hospice care

## Abstract

**Background:**

COVID-19 has tragically resulted in over 2.5 million deaths globally. Despite this, there is a lack of research on how to care for patients dying of COVID-19, specifically pharmacological management of symptoms.

**Aim:**

The aim was to determine the dose ranges of pharmacological interventions commonly used to manage symptoms in adult patients dying of COVID-19, establish how effectiveness of these interventions was measured, and whether the pharmacological interventions were effective.

**Design:**

This was a rapid systematic review with narrative synthesis of evidence, prospectively registered on PROSPERO (ID: CRD42020210892).

**Data sources:**

We searched MEDLINE, EMBASE, CINAHL via the NICE Evidence Health Databases Advanced Search interface; medRxiv; the Cochrane COVID-19 Study Register; and Google Scholar with no date limits. We included primary studies which documented care of patients dying of COVID-19 under the care of a specialist palliative care team.

**Results:**

Seven studies, documenting the care of 493 patients met the inclusion criteria. Approximately two thirds of patients required a continuous subcutaneous infusion with median doses of 15 mg morphine and 10 mg midazolam in the last 24 h of life. Four studies described effectiveness by retrospective review of documentation. One study detailed the effectiveness of individual medications.

**Conclusions:**

A higher proportion of patients required continuous subcutaneous infusion than is typically encountered in palliative care. Doses of medications required to manage symptoms were generally modest. There was no evidence of a standardised yet holistic approach to measure effectiveness of these medications and this needs to be urgently addressed.

**What is already known**

- COVID-19 has a mortality of between 1% and 2% and is the deadliest pandemic in living memory.
- The elderly, and those with pre-existing conditions tend to be most vulnerable to severe disease and death. Common symptoms experienced at the end of life include breathlessness and agitation/delirium.
- Care of those dying of COVID-19 is an understudied aspect of the pandemic.

**What this paper adds**

- This paper is the first review of international studies describing pharmacological symptom management of adult patients dying of COVID-19.
- Our thorough search found only seven papers that documented pharmacological symptom management of this patient cohort, highlighting the lack of research in this area.

**Implications for practice, theory or policy**

- A higher proportion of patients required continuous subcutaneous infusions for medication delivery than is typically seen at the end of life.
- Modest doses of commonly used end of life medications were required for symptom control.
- There was a lack of information about how effectiveness was measured, and whether medications used effectively alleviated symptoms.

## Introduction

The COVID-19 pandemic is the most significant public health crisis in living memory, and over 2.5 million people have tragically lost their lives.^1^ Mortality rates vary by age and comorbidities but are in the region of 1%–2%.^2^ To date, most of the pharmacological research has focused on drug treatments aiming for cure, amelioration of disease (cutting down the days of illness) or prevention (vaccines) with remarkable breakthroughs.^3,4^ Pharmacological symptom management for patients dying of COVID-19 is deserving of the same rigorous research approach and financial support. For those numerous individuals who are severely ill and symptomatic, there is additional need for expertise on symptom control and the impact of symptoms on health-related quality of life and the dying phase. A number of palliative care teams have published details of their care of patients dying of COVID-19. However, to our knowledge, this information has yet to be analysed and distributed to palliative care teams internationally. We aim to establish the dose ranges of pharmacological interventions commonly used to manage symptoms in adult patients dying of COVID-19. Secondly, to understand how health care professionals evaluate the effectiveness of pharmacological interventions in this cohort. Finally, to determine whether these pharmacological strategies were effective.

## Methods

There is lack of a shared definition for a rapid review.^5^ Common themes identified to define a rapid review in comparison to a systematic review include: accelerated timeline, streamlining or omission of methods, limited scope and resource constraints. This study shared many of these characteristics. Our scope was limited and clearly defined. For example, we did not consider oxygen therapy, or symptom relief by managing infection. We conducted this review in a short time frame, due to clinical need (the review was registered on PROSPERO in October 2020). Whilst we conducted a thorough search strategy, grey literature was not searched and there was an English language restriction. Despite these constraints, duplication of screening and extraction was maintained to enable confidence, and reduce potential bias in our results. Covidence software was used throughout to expedite the review process, with tables generated in Microsoft Excel.

### Literature search

This review was registered with PROSPERO (ID: CRD42020210892)^6^ and conducted over a 4-month period from October 2020 – January 2021. We searched the following databases from inception to the search date (09/10/2020): MEDLINE, EMBASE, CINAHL via the NICE Evidence Health Databases Advanced Search interface; medRxiv; the Cochrane COVID-19 Study Register; and Google Scholar. The search strategies used text words and relevant indexing to capture papers about the symptoms of palliative patients with COVID-19, using an adapted version of the COVID-19 search strings available from Public Health England.^7^ We searched for papers in English only, with no date limit. We also screened the bibliographies of the included full text articles. Our full search strategy is available as supplementary material.

### Eligibility criteria

#### Population

We included patients dying of COVID-19 infection, who received at least one review from the specialist hospital palliative care team, where a diagnosis of COVID-19 was made either on nasopharyngeal swab positive for SARS-CoV-2, or classic radiological evidence of disease. We excluded pregnant patients, patients under 18 years of age, patients taking opioids who did not have a life-limiting disease, patients in hospital who did not have contact with the hospital palliative care team, patients who died in the community, and patients who died in the intensive care setting. Patients in the intensive care setting were excluded due to presence of disease modifying interventions for example, tracheal intubation for invasive mechanical ventilation, which in the authors’ experience, alters the way symptom management medications are used.

#### Intervention and control

We included studies that documented the use of palliative care medications in our population of interest, considering all routes of administration. We excluded non-pharmacological interventions and pharmacological intervention guidelines. There was no control group available for this review.

#### Outcomes

We aimed to calculate the mean, median, modal, range and interquartile range of medication doses administered in the last 24 h of life. However, we were limited by the differences in the way data was presented between the studies, and were only able to report the median dose. The doses of medication were recorded in milligrams (mg), or micrograms (μg) in the case of glycopyrronium. In order to facilitate this analysis, we converted opioids to their oral morphine equivalent using established clinical conversions.^8^ We explored how authors documented effectiveness of pharmacological interventions in their patient cohort. We determined the effectiveness of these treatments, by looking for any formal, documented measure of effect.

### Study selection

The titles and abstracts of the studies retrieved during the searches were reviewed by two authors (LH and AL). Any disagreements over inclusion/ exclusion were referred to MM and MC. Studies considered potentially eligible for inclusion were assessed as full text articles by MC and MM with a third author (AL) deciding any disagreements. Data extraction was conducted by three authors (LH, MM, and MC), and checked by AL who was not involved in the initial extraction. Each included study was discussed by the research team for relevance, potential bias and rigour. We considered, but did not undertake, formal quality assessments for this rapid review, recognising that the small number of included studies were rapidly produced during the COVID-19 pandemic, and so of relatively poor quality. There are limited standardised assessment tools available for the types of studies (retrospective surveys, local audits) included in this review.

## Results

**Figure 1.**
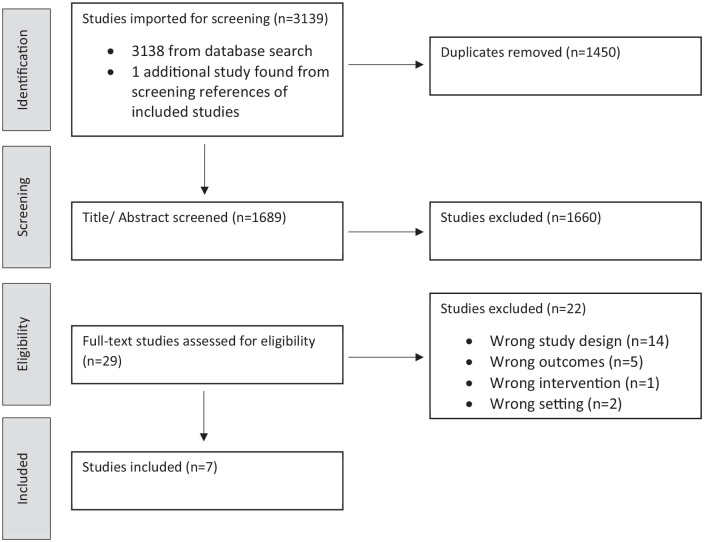
PRISMA flow diagram for included studies. In outline, these studies included a total of 493 patients from the UK (*n* = 6),^9–14^ and US (*n* = 1).^15^ Table 1 describes the key characteristics of each included study.

The search returned 3139 studies. Twenty-nine studies were reviewed at the full text stage and six were included in the review.^9–14^ One further study was identified when reviewing citations from these papers,^15^ resulting in a total of seven studies in this rapid review. The PRISMA diagram below (Figure 1) illustrates the attrition at each stage.

**Table 1.** Characteristics of included studies.

Author	Country	Aim/s	Study design	Date conducted	Setting	Inclusion criteria	Exclusion criteria
Alderman et al.^9^	UK	To assess the utility of our end-of-life care plan, and specifically the effectiveness of our standardised end-of-life care treatment algorithms, in dying patients with COVID-19.	Clinical audit	16/03/20 – 11/05/20	Inpatients in a medium sized NHS general hospital	1. All patients who died within the hospital with COVID-19 and had an end-of-life care plan.	1. 6 patients who survived and had an end-of-life care plan 2. 2 patients who were transferred elsewhere.
Heath et al.^10^	UK	To document the demographics, symptoms experienced, medications required, effectiveness observed, and challenges to high-quality holistic palliative care.	Retrospective survey	21/03/20 – 26/04/20	Inpatients at a tertiary NHS hospital in Oxford	1. Confirmed laboratory diagnosis of COVID-19 via reverse transcription polymerase chain reaction (RT-PCR) nasopharyngeal swab for SARS-Cov-2 and/or radiological evidence of coronavirus infection (commonly a plain chest radiograph reported as COVID -19 by a radiologist). 2.Died 3. Referred to HPCT	Not described
Hetherington et al.^11^	UK	1. To further characterise the symptom profile, outcomes and symptom management requirements of hospitalised patients with COVID-19 referred to Hospital Palliative Care. 2. To contextualise Palliative Care demands from COVID-19 against a ‘typical’ Palliative Care caseload pre-pandemic	Retrospective service evaluation compared with same seasonal period the previous year prior to the pandemic	30/03/20- 26/04/20	A large Scottish health board comprising 5 Hospital Palliative Care Teams and four acute receiving hospitals	1. Inpatients 2. RT-PCR nasopharyngeal swab positive for COVID-19 3. Referred to Hospital Palliative Care Team	Not described
Jackson et al.^12^	UK	1. To review whether prescribing for end-of-life symptom control for breathlessness, pain and agitation adhered to current regional standards 2. Where prescribing deviated from current guidance, to establish why this was necessary and whether different guidance for starting doses of symptom control drugs may be required for COVID-19 patients	Clinical audit	03/20 – 04/20	Patients in a large NHS teaching hospital in North East England	1. Inpatients referred to the palliative care team 2. Swab positive for SARS-CoV-2 3. Died during 1-month period March/April 20	1. Died before review by PCT 2. Dying phase not identified prior to death 3. Supported by ventilation on critical care unit 4. Background opioid prior to identification of the dying phase
Lovell et al.^13^	UK	To describe the symptom burden, management, response to treatment, and outcomes for patients with COVID-19 referred to palliative care.	Case series	04/03/20 – 26/03/20	Inpatients at two large acute NHS Hospital Trusts in London	1. Inpatients 2. Confirmed COVID infection 3. Referred to the hospital palliative care team	Not described
Sun et al.^15^	US	To describe the characteristics and palliative care needs in patients admitted to the Palliative Care Unit (PCU) in order to inform other clinicians caring for this population at the end of life	Case series	31/03/20 – 10/04/20	Newly created PCU in New York	1.Surrogates opted for comfort directed care 2. Admitted to Palliative Care Unit 2. Patients who died 3. Diagnosis of COVID-19 by PCR testing of nasopharyngeal swab	Not described
Turner et al.^14^	UK	1. Review the challenges faced during. this pandemic 2. Examine whether any patterns are arising, including unanticipated ones, to inform future care pathways	Case series from audit data	15/03/20 – 11/04/20	Inpatients at two acute NHS trust sites	1. Died 2. Positive RT-PCR nasopharyngeal swab for SARS-CoV-2 3. Inpatient	In critical care

Despite different methodological descriptions, all studies retrospectively gathered data from patient records who were at the end-of-life and diagnosed with COVID-19. Two studies were described as audits, and aimed to assess their care against established local standards or guidance for patients dying of other conditions.^9,12^ Details were not given on what these standards were, or how they were developed, which would have helped understand how their prescribing and symptom assessment differed (if at all) from usual practice. In contrast, another study (described as a case series from audit data) compared their continuous subcutaneous infusion (CSCI) use in COVID-19 (77%) to results in previous audits (33%) and discussed how medication doses used were comparable between COVID-19 and other diseases.^14^

Formal quality assessments were not attempted due to the abridged, pragmatic and informal methods the studies used. However, three main sources of bias were identified by the authors. Selection bias may have occurred if patients with challenging symptom management were differentially referred to the hospital palliative care team. This could result in over-estimation of medication doses required. Secondly, as medical practitioners may be influenced by local guidelines or practice, this could lead to observer bias when evaluating symptom control, or making prescribing decisions. Lastly, centres with more frequent symptom reviews may have a detection bias and be more likely to titrate up medications, resulting in higher end doses than other patient groups received. Together, these studies represent a small body of evidence, collected at a time when clinical services were under extreme pressure. All studies had been appropriately registered at their organisations and acknowledged limitations of their conclusions.

### What dose ranges of pharmacological interventions are commonly used to manage symptoms in patients dying of COVID-19?

Due to the difference in how drugs and dosing were reported, we calculated the number of patients receiving a CSCI (315, 64%) and median dose of morphine used in a CSCI in the last 24 h of life to be 15 mg subcutaneous morphine equivalent, and midazolam 10 mg subcutaneous (Table 2). The median dose of opioid equated to 30 mg of oral morphine equivalent.^8^

**Table 2.** Details of medications to manage symptoms given to patients dying of COVID-19 by Continuous Subcutaneous Infusion (CSCI) during the last 24 h of life.

Study	Number of participants	Number CSCI (%)	Number receiving opioid CSCI	Final dose morphine equivalent CSCI (median, mg)	Number receiving midazolam CSCI	Final dose midazolam CSCI (median, mg)
Alderman et al.^9^	61	41 (67%)	21	15	7	15
Heath et al.^10^	31	21 (68%)	Not described	10*	Not described	10*
Hetherington et al.^11^	186	140 (75.3%)	133	15	125	10
Jackson et al.^12^	48	33 (69%)	26	11.25	20	10
Lovell et al.^13^	101	58 (57%)	37	10	50	10
Sun et al.^15^	30	Not described	Not described	48	Not described	Not described
Turner et al.^14^	36	22 (72%)	Not described	15.96**	Not described	13.3**
Totals	493	315 (64%)	217	15***	202	10***

* Median of 21 patients who had CSCI, including those with 0mg morphine/ midazolam.

** Mean as median not described. Unclear whether this includes PRN doses.

*** Sun et al. and Turner et al. excluded as insufficient information to include in analysis.

Other medications used less frequently included haloperidol, levomepromazine, hyoscine butylbromide, glycopyrronium, cyclizine and metoclopramide. Three studies described the use of haloperidol.^9,11,13^ Two described a similar median dose and range of (2 (1–2) mg/24 h^13^ and 1.75(1–2) mg/24 h (*n* = 4)),^11^ whilst another study described higher doses of 5 mg/24 h (*n* = 4) and 10 mg/24 h (*n* = 3).^9^ Two studies described the dose of levomepromazine in a CSCI.^9,11^ One described a median dose of 15 mg/24 h (*n* = 16),^11^ and another a final dose of 75 mg/24 h (*n* = 14) and 150 mg/24 h (*n* = 2).^9^ The median dose and range of hyoscine butylbromide was described in one study as 60(40–120) mg (*n* = 21).^11^ The median dose and range of glycopyrronium was 1200 (600–2400) μg in one study,^13^ and 600 (600) μg (*n* = 8) in another.^12^ Further detail on cyclizine and metoclopramide doses were not given due to the small numbers of patients receiving these medications.

Authors reported the use of as needed medication, ‘Pro Re Nata’ (PRN) in several ways. One reported that ‘morphine intravenous equivalent bolus’ as a median of 3.3 mg.^15^ Another, that twenty-eight of thirty-three with a CSCI required PRN opioids (median 2.5 doses, median of 8.75 mg morphine equivalent) and 27 of 33 required PRN midazolam (median two doses, median 5 mg).^12^ A further study reported that of those not requiring a CSCI, 10% required at least one opioid PRN and 60% at least one PRN of midazolam; of those on an infusion 50% required at least one opioid PRN and 12% one PRN of midazolam.^9^ Two authors assessed that PRNs were prescribed.^10,13^ Two authors did not describe PRN medication data.^11,14^

### How are health professionals evaluating the effectiveness of pharmacological interventions used to manage symptoms in this cohort, and were they effective?

Four studies described how they judged effectiveness of pharmacological symptom management.^9–11,13^ One of these studies described the effectiveness observed with each medication prescribed in a 4-h assessment of common end-of life symptoms by the ward nurse.^9^ In this study, of the patients who were started on a CSCI for breathlessness, agitation and delirium, the majority of patients’ symptoms resolved at the first review point (4 h), and a minority of patients were still symptomatic at their last assessment before death (shortness of breath, 11.5%; agitation, 4.9%).^9^ Another study described how medications initiated were assessed as effective (78.6%) or partially effective (19%, required further dose titration) by a palliative medicine specialist during subsequent review of symptoms.^11^ In two studies, there was documented evidence of clinical effectiveness e.g. improved breathing, agitation or comfort in 69% of CSCIs,^13^ and 50% of PRN medications.^10^ Full medication and effectiveness data can be found in supplementary tables.

## Discussion

### Main findings

There is a lack of published data focusing on care at the end of life in this COVID-19 pandemic. We found a total of seven studies describing 493 patients where pharmacological management at the end-of-life was evaluated. To date, there have been over 2.5 million deaths from COVID-19,^1^ which equates to just 0.0002% of patients who died of COVID-19 have been represented in this literature. The studies included in this review were from the UK (*n* = 6),^9–14^ and the US (*n* = 1),^15^ where there have been 125,926 and 532,971 deaths respectively.^1^ The quantity and quality of published data does not reflect the scale of death in this pandemic. This finding in itself is important, as previous studies have highlighted the need for empirical evidence to justify current COVID-19 palliative care national and international guidelines.^16^

These collective results from seven studies suggest that relatively low doses of symptom control medications are required at the end of life for patients dying of COVID-19. The majority of patients received single or two drug combinations in their CSCI, most commonly morphine and/or midazolam. Although some patients did not require a CSCI, the proportion of patients (62%) that received a CSCI was approximately twice as high as reported in non-COVID-19 palliative care.^17^ However, these findings should be interpreted with caution, given the paucity of data with respect to the effectiveness of medications used to establish symptom control. Due to the small number of patients receiving haloperidol, levomepromazine, hyoscine butylbromide, glycopyrronium, metoclopramide and cyclizine, we were unable to specifically comment on the effectiveness of these medications. Similarly, extracting meaningful conclusions from the 78 patients with PRN medication data was not possible due to differences in reporting between studies.

### Results in context

These results are in line with existing international palliative care guidelines. The Worldwide Hospice Palliative Care Alliance (WHPCA) and International Association for Hospice and Palliative care (IAHPC) recommend low dose opioids and benzodiazepines to treat COVID-19 ‘if dyspnoea persists despite optimal treatment of the acute disease’.^18^ Starting doses in the guidance are similar to those described in this review. Rapid escalation of medication in line with symptom intensity is also supported, and prescribing PRN ‘rescue medication’ to be given up to hourly. Similarly, the UK National Institute of Clinical Excellence (NICE) recommends 10 mg/24 h morphine and 10 mg/24 h midazolam as starting dose for CSCI in those with symptomatic breathlessness from COVID-19 in the last days and hours of life.^19^ Our results provide some empirical evidence for these recommendations.

### Strengths and weaknesses of the study

A strength of this study is the robust study design, which was prospectively registered and published on PROSPERO.^6^ We did a comprehensive search of the literature in collaboration with an experienced librarian. Our flexible and pragmatic narrative synthesis allowed different metrics to be compared and discussed. To our knowledge this is the first attempt to collate information about the clinical management of dying in COVID-19.

Weaknesses of the study include not contacting authors for further clarification of their data. Due to the pace of the COVID-19 pandemic, and rapidly increasing mortality across the world, this was seen as a way to expedite the process of this rapid review. As our search was limited to studies published in English, we acknowledge there may be an Anglocentric bias in our results, which may not reflect international practice more broadly. Reassuringly however, our results are in line with international guidance.^16,18^ We include details of medication which we recognise may not be available in all parts of the world, and findings may need to be adapted to local pharmacological and health system constraints. Lastly, our review is only as robust as the included studies and there are significant methodological limitations in these studies. This is a reflection of the crisis situation experienced in palliative care during the pandemic, and the existing challenges of symptom management effectiveness assessments. Despite this, we believe there is value to taking stock of the current evidence, highlighting both areas of confidence, and areas that require future research.

### Limitations of included studies

Within the seven studies, weaknesses include lack of clarity about route of administration for example, whether medications were given orally, subcutaneously, as a CSCI or PRN, and few studies described the conversion ratios they used to calculate their drug doses. One study was an outlier in terms of high dose of opioids prescribed.^15^ Of note, patients included in this study were transferred to a specialist palliative care unit, and spent an average of 1.4 days in the unit before death. Further conclusions about the cause of comparatively high opioid doses are speculative and beyond the scope of this rapid review. A range of opioids were used in most studies, commonly morphine, oxycodone, fentanyl and alfentanil, but reasons for opioid selection such as renal and hepatic dysfunction or unacceptable adverse effects were not well described. One study described the estimated Glomerular Filtration Rate (eGFR) and level of chronic kidney disease at presentation with 3.8% of patients having an eGFR of <15 ml/min.^11^ Another reported a median eGFR of 67 ml/min (range 14–90) in their cohort.^12^ One study reports using only morphine for symptom management and noted that 18% of patients had background renal disease.^9^ Due to the risk of accumulation of clinically relevant active metabolites in renal impairment, this may explain the higher doses of sedatives given to patients in this study.^9^ Clarity regarding hepatic and renal function, route of administration and conversion ratios will be helpful in future studies to allow comparison and aggregation of datasets to guide best practice.

Similarly, there was lack of consistency between studies in how the spread of data was described. Most authors reported the median dose of the medications administered,^9–13,15^ with one group reporting the mean.^14^ Whilst ranges were given, no studies reported the modal dose administered for symptoms. This metric would be useful to report in the future to help understand the most frequent doses required to achieve symptom control.

### Challenges and recommendations

Robust effectiveness analysis of pharmacological interventions was challenging as only four studies commented on this.^9–11,13^ The World Health Organization defines palliative care as ‘an approach that improves the quality of life of patients and their families facing the problems associated with life-threatening illness, through the prevention and relief of suffering by means of early identification and impeccable assessment and treatment of pain and other problems, physical, psychosocial, and spiritual’.^20^ Impeccable or forensic symptom assessment is emphasised within this definition as it is increasingly recognized that palliative medicine must be tailored to the individual and that a ‘one size fits all’ approach is not conducive to quality end of life care.^21^ The assessment, treatment and re-assessment of symptom control in this group is challenging in general as the severely ill or disabled can find it difficult to contend with validated measures of symptom burden or health-related-quality of life. For this reason, abbreviated measures of both symptom burden and Health Related Quality of Life have been specifically developed and validated for use in this patient population. Furthermore, these are validated for completion by healthcare workers as proxy measures of the effectiveness of palliative treatments.^22^ In circumstances where patients are unconscious, tools such as The Respiratory Distress Observation Scale are validated and can identify when respiratory distress could benefit from as-needed intervention(s) in those who cannot report dyspnoea.^23^

The usual issues with symptom assessment in the palliative population are further compounded by the current pandemic, where palliative care is delivered at scale and in circumstances where health care workers are themselves under threat.^24^ Comprehensive and uniquely structured assessment of patients at the end of life requires skill and there are unavoidable issues of cultural context and patient-centred variables. The traditional hospice setting is designed to cater for this holistic approach but the hospital setting is expected to deliver intimacy at scale and in an environment where there are many competing priorities, leading to the limited use of robust iterative symptom assessment outside of the specialist hospice. In the busy hospital setting, family members keeping a vigil by a patient’s bedside can be invaluable assessors of the effectiveness of interventions aimed at symptom control. The isolation of the dying patient coupled with staffing crises will have further limited the capacity of teams to fully assess the impact of their interventions. Although our results show there is a need to implement a standardised method of reliably assessing response to medication, the unique challenge of the pandemic is acknowledged. Furthermore, the authors recognise that that symptom control is not a purely pharmacological endeavour and that quality holistic care can be difficult to measure.

Nevertheless, these studies highlight the need for a national and international consensus for a basic symptom assessment tool, that can be used in the crisis palliative medicine setting. The WHO described the vital role of palliative care in crisis settings, to include the development of a protocol for minimum standard of symptom assessment and treatment.^25^ This would enable clinical practice to be compared between medical centres and across borders at pace. Without this, individual centres operate in silos, changes in practice occur slowly, and as this review has found, effectiveness of interventions are difficult to interpret collectively.

### What this study adds

This is the first international review of pharmacological strategies to manage symptoms of patients dying of COVID-19. This review revealed an astonishing paucity of data, with only 493 patients having information about medication use at the end-of-life care documented and published, despite over 2.5 million recorded deaths worldwide.

We found that collectively across most published studies, low doses of commonly prescribed medications (morphine and midazolam) were used to manage the most prevalent symptoms of breathlessness and agitation.^26^ Despite low doses of medication, a higher proportion of patients required CSCI delivery, with two thirds of patients receiving medications this way. This is important to be aware of for care planning in hospitals and the community.

The quality of data reported reveals challenges in the way palliative care is delivered during COVID-19. We struggled to compare doses of medication used due to differences in reporting between studies, and lack of detail given on clinical conversions used between routes of administrations and different opioids. Similarly, effectiveness assessments were only fully described in one study.^9^

In conclusion, data on pharmacological management of end-of-life symptoms of COVID-19 is sparse. Seven studies that retrospectively reviewed patient notes were found following a thorough search. These studies show a greater use of continuous subcutaneous infusions, with modest doses of commonly used drugs. This data may serve as a barometer for palliative care departments, non-specialist inpatient settings and community care homes who are also managing these patients. The lack of information about assessment of effectiveness limits understanding of the data. An international consensus on how to assess effectiveness of symptom control at end of life in this cohort with unique challenges to care is required.

## Supplemental Material

sj-docx-1-pmj-10.1177_02692163211013255 – Supplemental material for Pharmacological strategies used to manage symptoms of patients dying of COVID-19: A rapid systematic review Click here for additional data file. Supplemental material, sj-docx-1-pmj-10.1177_02692163211013255 for Pharmacological strategies used to manage symptoms of patients dying of COVID-19: A rapid systematic review by Laura Heath, Matthew Carey, Aoife C Lowney, Eli Harriss and Mary Miller in Palliative Medicine
