# Supplementary material for: Pharmacological strategies used to manage symptoms of patients dying of COVID-19: A rapid systematic review
Source: Palliat Med. 2021 May 13;35(6):1099–107. doi: 10.1177/02692163211013255 (PMC8189007; doi:10.1177/02692163211013255)
Supplement: sj-docx-1-pmj-10.1177_02692163211013255 – Supplemental material for Pharmacological strategies used to manage symptoms of patients dying of COVID-19: A rapid systematic review [file sj-docx-1-pmj-10.1177_02692163211013255.docx]

Supplementary Table 1. Question 1: What dose ranges of pharmacological interventions are commonly used to manage symptoms in patients dying of COVID-19?

| Study | Number of participants | Number receiving CSCI | Details of drugs/ doses in CSCI | Number receiving PRNs | Details on drugs/ doses PRNs |
| --- | --- | --- | --- | --- | --- |
| Alderman et al. | 61 | 41 (67%) Symptom control: n=38 Maintenance antiepileptic medication: n=3 | Management of breathlessness:  n = 14 (34.5%) morphine  n = 7 (11.5%) morphine and midazolam  Initial dose morphine – 10mg/24 h (n=12); 15mg/24 h (n=9)  Final dose morphine – 10mg/24 h (n=10); 15mg/24 h (n = 10); 20mg/24 h (n=1)  Initial dose midazolam – 10mg/24 h (n=2); 15mg/24 h (n=5)  Final dose midazolam – as above (no change)  Management of agitation/delirium:  n = 24 (39.5%)  Haloperidol (first-line treatment):  Final dose – 5mg/24 h (n=4; initial dose); 10mg/24 h (n=3; dose titrated)  Levomepromazine (second-line treatment):  Final dose – 75mg/24 h (n=14; initial dose);150mg/24 h (n=1; dose titrated)  Levomepromazine and midazolam (third-line treatment):  Final dose – 150mg/24 h and 20mg/24 h, respectively (n=1)  Midazolam (first-line treatment): Final dose – 20mg/24 h | Management of breathlessness:  20 patients on CSCI needed at least one PRN dose of morphine, but only 2 patients required an increase in the dose of morphine in the syringe pump.  9 (15%) other patients needed at least one PRN dose of morphine, but were not started on regular morphine.  5 patients on CSCI needed at least one PRN dose of midazolam, but no patients required an increase in the dose of midazolam in the syringe pump.  12 (19.5%) other patients also needed at least one PRN dose of midazolam, but were not started on regular midazolam  Management of agitation/delirium:  Levomepromazine was initiated after primary failure to respond to PRN haloperidol in 11 patients and after secondary failure (initial response) to regular haloperidol in 5 patients.  Patient was treated with midazolam because of coexistent myoclonus.  5 (8%) other patients also needed at least one PRN dose of medication for agitation, but were not started on regular medication for agitation. | Drugs used include:  Morphine Midazolam Haloperidol  Levomepromazine  Glycopyrronium   No information on doses of PRNs used. |
| Heath et al. | 31 | 21 (68%) | The most frequent combination of medication administered was an opioid (morphine, oxycodone, or alfentanil) and a benzodiazepine (midazolam).  Doses were low with a median equivalent to 10mg morphine SC/24 hours and midazolam 10mg/24 hours.  Morphine was the opioid of choice in 71%, oxycodone in 19%, and alfentanil in 10%. | 18 (58%) | 100% had an opioid prescribed (morphine or oxycodone)  100% had midazolam prescribed.  90% had medication for delirium - haloperidol first line with 32% as levomepromazine second line.  94% had an antisecretory medication (Hyoscine butyl bromide)  61% had an antiemetic. |
| Hetherington et al. | 186 | 140 (75.3%) | Opioid and midazolam n=121.  Opioids: median daily subcutaneous morphine equivalent final dose 15mg [IQR  10,20] (range 5-90).  Midazolam: median final daily dose was 10 mg [IQR 10,20] (range 2.5–60).  CSCI pumps were stopped in seven cases due to improvement in clinical condition.  Morphine & midazolam n=62  Alfentanil & midazolam n=19  Morphine, midazolam & hyoscine butylbromide n=10  Oxycodone & midazolam n=9  Morphine, midazolam & levomepromazine n=7 Alfentanil & midazolam & haloperidol n=4  Alfentanil, midazoalm & hyoscine butylbromide n=3  Oxycodone, midazoalm & hyoscine butylbromide n=3  Oxycodone, midazoalm & hyoscine butylbromide n=3  Alfentanil alone n=3  Morphine alone n=3  Morphine, midazolam, levomepromazine & hyoscine butylbromide n=2  Midazolam alone n=2  Morphine & metoclopramide n=1  Alfentanil & metoclopramide n=1  Morphine & levomepromazine n=1  Alfentanil, midazolam, levomepromazine & hyoscine butylbromide n=1  Alfentanil, levomepromazine & hyoscine butylbromide n=1  Morphine & hyoscine butylbromide n=1  Alfentanil, midazolam & metoclopramide n=1 Levomepromazine alone (n=1  Drug info not available n=2  Drug dose in 24 hours; median, (range) [IQR]  All opiates in sub cut morphine equivalent (n = 133) 15 mg (5–90) [10, 20]  Morphine (n = 87)15 mg (5–90) [10, 20]  Oxycodone (n = 15)10 mg (5–40) [8, 17.5]  Alfentanil (n = 33)900 mg (300–4000) [500, 1000]  Midazolam (n = 125)10 mg (2.5–60) [10, 20]  Haloperidol (n = 4)1.75 mg (1–2)  Hyoscine butylbromide (n = 21) 60 mg (40 120) Levomepromazine (n = 16) 15 (100)?unclear | Not clear - presented as total dose in 24 hours | Not described |
| Jackson et al. | 48  But subgroup analysis of 42 (last 24 hours of life) | 33 (69%) | 1. Opioid n=26   Mean: 11.9mg (range: 5–45mg) Median: 10mg   1. Midazolam n=20   Mean: 10.5mg (range: 5–25mg) Median: 10mg   1. Glycopyrronium n= 8   Mean: 600μg (range: 600μg) Median: 600μg | 1. Opioid n=28 2. Midazolam n=27 3. Glycopyrronium n=19 | 1. Opioid: Number of doses: Mean: 2.5 (range: 1–7) Median: 2.5  Dose requirements: Mean: 9.4mg (range: 2–35mg) Median: 8.75mg  2. Midazolam: Number of doses: Mean: 2.1 (range: 1–6) Median: 2  Dose requirements: Mean: 7.0mg (range: 2.5–25mg) Median: 5mg  3. Glycopyrronium: Number of doses: Mean: 1.2 (range: 0–2) Median: 1  Dose requirements: Mean: 242μg (range: 200–400μg) Median: 200μg |
| Lovell et al. | 101 | 58 (57%) | Of the 37 patients who were prescribed morphine by subcutaneous infusion, the median final dose was 10mg/24 hours. Fifty infusions contained midazolam, median final dose 10mg/24 hours.  Median (range) dose/24 hours Morphine 10 (5 - 30)mg Fentanyl 100 (100 - 200)μg Alfentanil 500 (150 - 1000)μg Midazolam 10 (10 - 20)mg  Glycopyrronium 1200 (600 - 2400)μg Haloperidol 2 (1 - 2)mg Cyclizine 50mg  *(No comment as to whether a combination  Of PRNs, CSCIs or both) | Ninety-six patients were prescribed ‚’as needed’ medication for symptom relief | Median (range) dose/24 hours Morphine 10 (5 - 30)mg Fentanyl 100 (100 - 200)μg Alfentanil 500 (150 - 1000)μg Midazolam 10 (10 - 20)mg  Glycopyrronium 1200 (600 - 2400)μg Haloperidol 2 (1 - 2)mg Cyclizine 50mg  *(No comment as to whether a combination  Of PRNs, CSCIs or both) |
| Sun et al. | 30 | Not described | Morphine IV equivalent infusion rate; median 2mg/ hour (Range: 1-6mg/hour) | Not described | Morphine IV equivalent bolus; median 3.3 mg (Range: 1.3-10mg) |
| Turner et al. | Deaths: n=36  Notes available: n=30 | 22 (72%) | Not described | Not described | Not described |

Supplementary Table 2. Question 1 continued: What dose ranges of pharmacological interventions are commonly used to manage symptoms in patients dying of COVID-19? (Last 24 hours)

| Study | Morphine equivalent | Benzodiazepines | Antipsychotics | Anticholinergics | Other |
| --- | --- | --- | --- | --- | --- |
| Alderman et al. | Final doses in CSCI - 10mg (10), 15mg (10) and 20mg (1) | Final dose Midazolam 10mg (2) and 15mg (5) | Haloperidol 5mg (4) and 10mg (3)  Levomepromazine 75mg (14) 150mg (1) | Glycopyrronium used but no doses given. | Antiemetics unknown |
| Heath et al. | The median total oral morphine equivalent dose in the last 24 hours of life (sum of syringe driver and ‘as required’ doses converted to oral equivalent) was 20mg [IQR 14-38mg]. | The median total dose of benzodiazepine (midazolam) was 10mg [4-15mg]. This equates to 20mg diazepam PO/24 hour | Not described | Not described | Not described |
| Hetherington et al. | All opiates in sub cut morphine equivalent (n = 133)15 mg (range 5–90) [IQR 10, 20]  Conversion of opioid dose to subcutaneous morphine equivalence was based on a conversion of 15:1 for alfentanil and 2:1 for subcutaneous oxycodone. | Midazolam (n=125) median final daily dose was 10 mg (range 2.5–60) [IQR 10,20]. | Haloperidol (n = 4)1.75 mg (range 1–2) | Hyoscine butylbromide (n = 21) 60 mg (range 40 - 120) | eGFR at presentation; n (%):  ⩾60 100 (53.8%)  45–59 30 (16.1%)  30–44 28 (15.1%)  15–29 21 (11.3)  <15 7 (3.8%) |
| Jackson et al. | Mean: 14mg (range: 0-55mg) Median: 11.25mg | Midazolam Mean: 9.5mg (range: 0-35mg) Median: 8.75mg | Not described | Gycopyrronium Mean: 196μg (range: 0–1000μg) Median: 0mg | Nausea not reviewed as not an important Covid symptom |
| Lovell et al. | Median (range) dose/24 hours Morphine 10 (5-30)mg  Fentanyl 100 (100-200)μg Alfentanil 500 (150-1000)μg | Median (range) dose/24 hours  Midazolam 10 (5-20)mg | Median (range) dose/24 hours  Haloperidol 2 (1-2)mg | Median (range) dose/24 hours Glycopyrronium 1200 (600--2400)μg | Median (range) dose/24 hours  Cyclizine 50mg |
| Sun et al. | Not described | Not described | Not described | Not described | Not described |
| Turner et al. | 26 (86%) received opioids in final 24 hours.  Total mean SC morphine equivalent dose in final 24 hours - 15.96mg | 24 (81%) received benzodiazepines in final 24 hours.  Total mean SC midazolam dose in final 24 hours 13.3mg | Not described | 44% received hyoscine Butylbromide in final 24 hours | Not described |

Supplementary Table 3. Question 2: How are health professionals evaluating the effectiveness of pharmacological interventions used to manage symptoms in this cohort? Question 3: Were the pharmacological interventions used to manage symptoms in this patient cohort effective?

|  | Question 2 | Question 3 | | | | |
| --- | --- | --- | --- | --- | --- | --- |
| Study | Describe how the study did this. | Opioids | Benzodiazepines | Antipsychotics | Anticholinergics | Other |
| Alderman et al. | With review of the ‘End of Life Care Plan’ initiated in the last week of life. This includes four-hourly assessment of common end-of life symptoms by the ward nurse. An in-house standardised treatment algorithm initiated if symptoms present. | 1. Pain was reported in 12 (19.5%) patients: only three patients were prescribed a regular analgesic (morphine) and only one patient had pain in their final assessment 2. 10 (62.5%) patients that were started on a continuous subcutaneous infusion of morphine at the initial assessment had relief of their shortness of breath within 4 h. Of the remaining patients, two (12.5%) patients died within 4 h of starting the continuous subcutaneous infusion, and two (12.5%) patients needed midazolam to treat shortness of breath-related anxiety. Similarly, shortness of breath was a problem for ⩽13%  patients in the last 72 h of life (range: 0% -13%) and for 11.5% patients at the final assessment before death | 1. Agitation / Delirium 14 (100%) patients who were started on a continuous subcutaneous infusion at the initial assessment had relief of their agitation within 4 h: 7 (50%) of these patients had no further episodes of agitation. Similarly, agitation was a problem for ‚⩽  11.6% patients in the last 72 h of life (range: 0% -11.6%) and for 4.9% patients at the final assessment before death (Figure 2). 2. Breathlesness two patients needed midazolam to treat shortness of breath-related anxiety.    Paradoxical agitation noted in 1 patient | 14 (100%) patients who were started on a continuous subcutaneous infusion at the initial assessment had relief of their agitation within 4 h: 7(50%) of these patients had no further episodes of agitation. Similarly, agitation was a problem for ⩽11.6% patients in the last 72 h of life (range: 0% -11.6%) and for 4.9% patients at the final assessment before death (Figure 2). | Death rattle was observed in 11 patients, with most episodes occurring in the last 12 h of life and most (10 out of 11) episodes not persisting beyond one assessment. In three cases, the problem improved without use of medication, while in eight cases medication was utilised (i.e. as required glycopyrronium in seven cases and regular glycopyrronium in one case). The patient with the persistent problem did not respond to glycopyrronium | Nausea was reported in two (3.5%) patients: only one patient was prescribed a regular anti-emetic (haloperidol). No patient had nausea (or vomiting) in their final assessment Fever - 1 person Cough - none |
| Heath et al. | Where the patient had received an ‚’as required’, medication, there was documentation of efficacy in 50% | Not described | Not described | Not described | Not described | Not described |
| Hetherington et al. | Data were extracted from medical notes, nursing notes, drug charts electronic records and referral systems by ward-based clinicians. Clinical impression of efficacy was deemed effective (symptoms improved, and no further titration required) or partially effective (improvement in symptoms but further titration advised) | Not described | Not described | Not described | Not described | 126 cases  Effective 99 (78.6%)  Partially effective 24 (19%)  Not effective 3 (2.4%)  7 cases where CSCI stopped due to clinical improvement |
| Jackson et al. | Unable to objectively assess whether symptom control was fully achieved. | Not described | Not described | Not described | Not described | Not described |
| Lovell et al. | Clinical impressions of effectiveness were determined based on documentation at follow-up (e.g. improved breathing, agitation, comfort). | Not described | Not described | Not described | Not described | Not divided into individual medications.  Clinical impression of effectiveness Yes: n=40 Unclear (patient died before follow up): n=13 No: n=5 |
| Sun et al. | Not described | Not described | Not described | Not described | Not described | Not described |
| Turner et al. | No comment on effectiveness | Not described | Not described | Not described | Not described | Not described |
